# Generating truth from error: insights from neurodevelopmental disorders

**DOI:** 10.1093/brain/awy311

**Published:** 2018-12-27

**Authors:** Ashwani Jha, Parashkev Nachev

**Affiliations:** Institute of Neurology, UCL, 33 Queen Square, London, UK

## Abstract

This scientific commentary refers to ‘Impaired forward model updating in young adults with Tourette syndrome’, by Kim *et al.* (doi:10.1093/brain/awy306).

This scientific commentary refers to ‘Impaired forward model updating in young adults with Tourette syndrome’, by Kim *et al.* (doi:10.1093/brain/awy306).

‘If only *one* person had, *once*, made a bodily movement—could the question exist, whether it was voluntary or involuntary?’ (Wittgenstein, 1980).

In the answer are revealed two incontestable features of the biology of voluntary action, incontestable because they are conceptually given and so impregnable to empirical attack. First, to be able to say of someone that she acted voluntarily we must be able to say that she could have acted otherwise even if, in the event, she did not. This implies a plurality of condition-movement associations—including the absence of movement—and a mechanism for selecting between them. A substrate that instantiates this, neurally or mechanically, can only be described as embodying a model, for that is what a set of rules of conditional transformation is. Second, to be able to act voluntarily one must be able to act as one has never acted before, for the learnt acquisition of any ability implies it must have been novel, once, and the ability here must be autonomously acquired. So, the model must be generative, capable of interpolating across the high dimensional space of condition-movement associations it has learnt.

How could models of this kind operate in the brain? Since their objective is to orchestrate action, they must be primarily optimized at the output, not any point upstream, for that is what they are deployed to shape. Such forward models compare their output estimates against reality as far as the sensorium is capable of disclosing it and then adjust to optimize future predictions (Wolpert and Ghahramani, 2000; Friston, 2018). With respect to kinematic features, the comparison might plausibly be a simple loss function based on the squared error of position; the more complex objective functions needed for teleological characteristics need not alter the basic principle of optimizing predictive fidelity. The model’s estimate cannot be altered experimentally, but the sensory signals it relies on for comparison can be, at least within modalities we can readily manipulate. Pathological defects of model optimization may thereby be revealed.

This is the approach Kim and co-workers (2019) elegantly apply, in this issue of *Brain*, to Gilles de la Tourette syndrome, a complex disorder characterized by prominent, polymorphous tics, whose frequent comorbidity with other neurodevelopmental disorders involving impaired behavioural control suggests a deep, shared mechanism of causation. Young adults with the syndrome were compared against controls on a double-step manual reaching task employing a robotic manipulandum designed to record reaching trajectories while controlling visual feedback. On each trial, participants reached from one of four ‘home’ positions to one of four ‘target’ positions, returning to their starting point as the second step. The movement was obscured by a screen on which both positions were briefly visible only at trial onset, leaving proprioception as the only feedback throughout. Spatial error was thus allowed to accumulate across the trial, measured at the target and home return terminations. Though just as accurate and no more variable on the outward leg, those with the syndrome were less accurate and more variable on the return. Crucially, the return movement was consistent with an updated model of its initial position, compensating to some degree for the error on the way out, but the extent of updating was attenuated compared with the control group. It seems Gilles de la Tourette syndrome is associated with reduced model updating in a sensorially deprived environment: error is detected, but the motor programme is not sufficiently updated in response.

Correlation with comorbid attention deficit hyperactivity disorder—though not tic scores—suggests this is not a narrowly motor defect. The authors propose the attractive idea that pathologically enhanced sensorimotor noise may force a reduction in the rate of model updating, for example because the loss function becomes less reliable. Indeed, when training artificial neural networks, the optimal learning rate is related to the scale of noise (Smith and Le, 2018). This plausibly general phenomenon may well explain some of the comorbid diversity of neurodevelopmental disorders.

The relation to the disturbed voluntariness of action in Gilles de la Tourette syndrome is harder to define. It is tempting to conceive of the model’s estimate as an ‘internal signal’ of the authorship and voluntariness of a movement: the more accurate the former, the stronger both of the latter (Ganos *et al.*, 2015). The same logic, deployed across individuals, is pursued by those who interpret mirror neuron activity as reflective of establishing a commonality between self and other. But authorship is distinct from voluntariness. We are never in any doubt that (say) a sneeze, a blink, a cough, or a yawn is our own, even though each is usually involuntary and need not be accompanied by an over-riding external trigger. Alien penis syndrome is not a recognized nosological entity despite our manifest lack of control over erectile dynamics. Equally, model accuracy is not a plausible index of voluntariness. The relative simplicity and stereotypy of involuntary movements will tend to render them accurately predictable. Since a novel voluntary action will naturally be associated with lower model accuracy than a well rehearsed one—the error, after all, is what drives learning—the absurd implication is that novelty and voluntariness must be inversely related. Moreover, erroneous sensory feedback may not only disrupt the correct attribution of voluntariness to a movement but also mistakenly attach it to an involuntary one, indeed—as in the ‘moving rubber hand illusion’ (Dummer *et al.*, 2009)—to no actual movement at all. Here the supposedly authoritative internal criterion is easily over ruled by an external sensory stimulus.

There is a deeper, information-theoretic objection. Empirical studies tend to focus on adaptation, in adults, after an action is already well formed. But most features of action cannot be genetically specified—there is nowhere near enough room in the genome (Nachev *et al.*, 2018)—and so must be learnt *de novo.* We must explain not only how model estimates are optimized but also how they are generated in the first place: the nature of the ‘trial’, not just of the ‘error’. Without loss of generality, we may conceive of our model as the result of the interaction between a generator that proposes a set of movement characteristics, and a discriminator that evaluates their goodness with respect to the objective. The generator must be initialized with more or less random noise—no other option is available—and such organization as it acquires can only come from its operation within the model. The discriminator is definitionally anchored in sensorially-conveyed external reality, and must integrate information across the typically vast spectrum of internal and external factors material to the shaping and selection of actions. Crucially, for such a mechanism to work neither component can dominate: if the generator does not yield to the discriminator, it definitionally cannot learn anything at all, and if the discriminator always rejects the generator, no new action could ever emerge. The interaction will not be the simple zero-sum game typically employed in (say) generative adversarial networks (Goodfellow *et al.*, 2014), but its nature cannot change the fundamental point: no isolated component of the model—and certainly not the generator—can be a ground truth of either authorship or voluntariness, for the information is constitutionally widely distributed.

Indeed, the idea of a purely internal neural signal is analogous to the idea of a purely private mental object, and fails for the same reason: an isolated representation can be neither created nor remain stable because the information to create and sustain it cannot arise (Wittgenstein, 1953). A private language and a private neural signal are equally—and information theoretically—impossible (Fig. 1).

**Figure 1:**
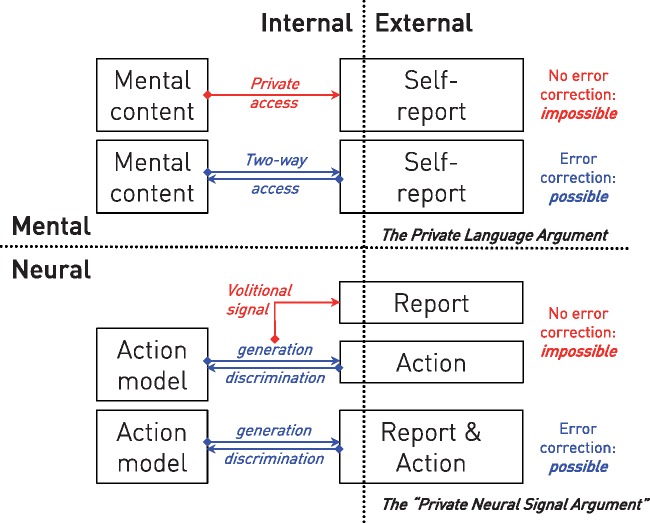
**The impossibility of private representations: mental or neural.** In what has become known as the Private Language Argument, Wittgenstein showed that a purely internal mental object can be neither created nor referenced because no criterion of correctness can be applied (*top*). This is analogous to what we might call the Private Neural Signal Argument where an internal neural signal alone cannot set the ground truth if it is itself created by error feedback (*bottom*).

The conceptual difficulties of voluntary action would not be so opaque were the field of motor control not so focused on adults. Attempting to understand the brain from its mature operations is rather like attempting to understand artificial neural networks only after extensive training. Their societal impact aside, neurodevelopmental disorders deserve much more intense study of the kind Kim and her colleagues exemplify, uniquely illuminating physiology from two intersecting angles: development and pathology. And that light will need amplifying with large scale data, for the complexity is here compounded by its wide dispersal over developmental age.

But we should also remember there are problems that cannot be solved, only dissolved. Wittgenstein may well be right that the ‘sense of agency’ is one of them:

‘But how do I know that this movement was voluntary? — I don’t know this, I manifest it.’ (Wittgenstein, 1980).

## Funding

A.J. and P.N. are funded by the Wellcome Trust and the UCLH NIHR Biomedical Research Centre.

## Competing interests

The authors report no competing interests.
